# Surgically Resected Esophageal Squamous Cell Carcinoma: Patient Survival and Clinicopathological Prognostic Factors

**DOI:** 10.1038/s41598-020-62028-5

**Published:** 2020-03-19

**Authors:** Dong Young Jeong, Kyung Soo Lee, Joon Young Choi, Myung Jin Chung, Yang Won Min, Hong Kwan Kim, Jae Ill Zo, Young Mog Shim, Jong-Mu Sun

**Affiliations:** 1 Department of Radiology, Samsung Medical Center, Sungkyunkwan University School of Medicine (SKKU-SOM), Seoul, South Korea; 2 Department of Nuclear Medicine, Samsung Medical Center, Sungkyunkwan University School of Medicine (SKKU-SOM), Seoul, South Korea; 3 Division of Gastroenterology, Department of Medicine, Samsung Medical Center, Sungkyunkwan University School of Medicine (SKKU-SOM), Seoul, South Korea; 4 Department of Thoracic Surgery, Samsung Medical Center, Sungkyunkwan University School of Medicine (SKKU-SOM), Seoul, South Korea; 5 Division of Hemato‐oncology, Department of Medicine, Samsung Medical Center, Sungkyunkwan University School of Medicine (SKKU-SOM), Seoul, South Korea

**Keywords:** Oesophageal cancer, Surgical oncology

## Abstract

We aimed to report patients’ survival after surgical resection of eSCC and to ascertain the clinical, imaging, and pathological factors related to patient prognosis. This retrospective study included 435 patients with eSCC of <stage T2 (median follow-up period, 49.3 months). A total of 103 (23.7%) patients died, and 89 (20.5%) experienced recurrence during follow-up. The maximum standardized uptake value (SUV_max_) on positron emission tomography (PET)/computed tomography (CT) of the primary tumor was significantly correlated with tumor length, nodal metastasis, and pathologic T stage in a positive linear fashion. In the multivariate analysis, higher SUV_max_ on PET/CT was a negative prognostic factor for both disease-free survival (DFS) and overall survival (OS). Contrarily, the presence of nodal metastasis was a prognostic factor only for DFS, and pathologic T stage only for OS. By applying SUVmax cut-off, both DFS and OS were significantly different among three groups when divided by cut-off values (A: SUVmax ≤ 3.05, B: SUVmax 3.06 - 5.64, C: SUVmax ≥ 5.65). In patients with a surgically resectable eSCC, measuring the SUV_max_ of the primary tumor during PET/CT can help predict patient survival. Additionally, PET/CT renders triage criterion for endoscopic submucosal dissection (ESD; T1a cancer and SUVmax, ≤3.05).

## Introduction

Esophageal cancer (ECA) is the eighth most prevalent cancer, accounting for 4% of all cancers. It is also the sixth leading cause of cancer-related deaths. Although the prevalence of esophageal adenocarcinoma is rapidly increasing in Western countries, including the United States of America, the United Kingdom, and Australia, the most common histopathologic subtype worldwide is esophageal squamous cell carcinoma (eSCC); it accounts for 85% of all esophageal cancers^1^.

The incidence of ECA is rapidly increasing. The overall 5-year survival rate ranges from 15% to 25%, with the best outcomes expected in ECAs diagnosed early^2^. In patients with an early-stage eSCC less than clinical T2 stage, treatment of choice is esophagectomy^3,4^. However, esophagectomy brings high risks of surgical complication and hospital mortality with incidences varying between 17–74% and 7–9%, respectively^5^.

In patients with a T1a eSCC, endoscopic resection such as endoscopic submucosal dissection (ESD) can be considered as an alternative treatment option to surgery with cure rates similar to those in esophagectomy and with low complication rate^3,4,6^. According to recent Japanese Esophageal Society Guidelines, relative indication of ESD is extended to some portion of T1b eSCC involving the muscularis mucosa or <200-μm invasion of the submucosa^7,8^. Thus, adequate and accurate staging should be performed to provide adequate treatment options to patients, especially in elderly patient who cannot tolerate surgical procedure.

Most clinical staging work-ups of ECAs are conducted using endoscopic ultrasonography (EUS), computed tomography (CT), and ^18^F-fluorodeoxyglucose positron emission tomography (FDG) PET/CT. In our previous study, we showed that the depth of tumor invasion (T stage) and the presence of LN metastasis (N stage) could be identified with FDG PET/CT even in early esophageal SCCs. However, the results are slightly inferior to those of EUS^9^. With the measurement of maximum standardized uptake value (SUV_max_) of the primary tumor at FDG PET/CT, the depth of tumor invasion (T stage) could be readily predicted.

Therefore, we hypothesized that measuring the SUV_max_ of the primary tumor in early-stage eSCCs could help predict patient prognosis. The purpose of this study was to report patients’ survival after surgical resection of eSCCs, including T1a-, T1b-, and T2-stage cancers, and to ascertain the clinical, imaging, and pathological factors related to patient prognosis.

## Results

### Patient characteristics and clinical outcomes

Of the 435 patients, 403 were men, and 32 were women. Their ages ranged from 31 to 90 years (mean, 64 years). One-hundred and thirty-one patients had less than T1a stage disease, 234 patients had T1b stage disease, and 70 patients had T2 stage disease. Primary tumors presented pathologically with N0 disease in 298 (68.5%) patients. The presence of nodal metastasis was confirmed with surgical specimens in 137 (31.5%) patients. N1 disease was identified in 99 (22.8%) patients, N2 in 32 (7.4%) patients, and N3 in six (1.4%) patients. Other patient characteristics are detailed in Table 1.

**Table 1:** Patient Demographics and Tumor Characteristics of the Experimental Group.

Characteristics	Pathologic stage				
	Tis + T1a	T1b	T2	Total	*p-*values*
Sex					0.608
Male	119	218	66	403	
Female	12	16	4	32	
Mean age (yrs)**	64	64	66	64	0.400
(min–max)	(31–90)	(40–85)	(44–78)	(31–90)	
Tumor length (cm)	2.07	2.29	3.40	2.36	**<0.001**
(Q1–Q3)	(1.20–2.55)	(1.40–2.85)	(2.50–3.85)	(1.50–3.00)	
Tumor differentiation					**0.020**
G1	26	31	11	68	
G2	96	169	43	308	
G3	9	34	16	59	
Adjuvant Tx					**<0.001**
Yes	4	53	20	77	
No	127	181	50	358	
R0 resection					0.602
Yes	130	231	70	431	
N0	1	3	0	4	
Tumor Locations					0.147
Cervical	0	1	0	1	
Upper thoracic	14	16	5	35	
Mid thoracic	50	99	20	169	
Lower thoracic	52	102	35	189	
Upper to mid thoracic^†^	3	6	0	9	
Mid to lower thoracic^†^	10	8	10	28	
Upper to lower thoracic^†^	1	1	0	2	
Upper and lower thoracic^††^	1	1	0	2	
Nodal metastasis					**<0.001**
No	121	146	31	298	
Yes	10	88	39	137	
Clinical T stage using EUS					**<0.001**
Less than T1a	79	76	3	158	
T1b	41	105	15	161	
T2	11	47	34	92	
T3	0	6	18	24	
PET staging using cut-off					**<0.001**
A: SUV_max_ < 3.05	8	9	1	18	
B: SUV_max_ 3.06–5.64	71	67	2	140	
C: SUV_max_ ≥ 5.65	41	105	15	161	
SUV_max_	2.53	4.02	9.69	4.48	**<0.001**
(Q1–Q3)	(1.00–3.10)	(2.50–4.80)	(5.78–13.4)	(2.40–5.40)	
Perineural invasion	0	0	7	7	**<0.001**
Angiolymphatic invasion	0	0	3	3	**<0.001**
Recurrence	16 (12.2%)	51 (21.8%)	22 (31.4%)	89 (20.5%)	**<0.001**
Death	23 (17.6%)	54 (23.1%)	26 (37.1%)	103 (23.7%)	**0.007**
Total	131	234	70	435	

Note. ^__^ *Calculated with Chi-square test, Q = quartile (Q1–25 percent quartile, Q3–75 percent quartile); G1 = well-differentiated, G2 = moderately differentiated, G3 = poorly differentiated; Definition of the tumor location: Cervical = superior to the thoracic inlet, Upper thoracic = from the thoracic inlet to the azygos arch, Middle thoracic = from the azygos arch to the inferior pulmonary veins, Lower thoracic = from the inferior pulmonary veins to the esophagogastric junction; Upper to middle thoracic^†^ = long segment involvement from the upper to middle esophagus, Mid to lower thoracic^†^ = long segment involvement from the middle to lower esophagus, Upper to lower thoracic^†^ = long segment involvement from the upper to lower esophagus; Upper and lower thoracic^††^ = the upper and lower thoracic esophagus skipping the middle thoracic esophagus.

EUS: Endoscopic ultrasonography; PET: positron emission tomography; SUV_max_: maximum standardized uptake value.

During surgical resection, four patients underwent R1 resection indicating the presence of microscopically cancer-cell-positive tumor margins. Seventy-seven patients underwent adjuvant therapy, of which 74 underwent chemotherapy, two underwent radiotherapy, and one underwent concurrent chemoradiation.

The median follow-up duration was 49.3 months (range; 0.8 to 105 months). One-hundred and three patients died, and 89 patients experienced recurrence during the follow-up period. Details of the relationship between death or recurrence and T stages are summarized in Table 1.

**Figure 1:**
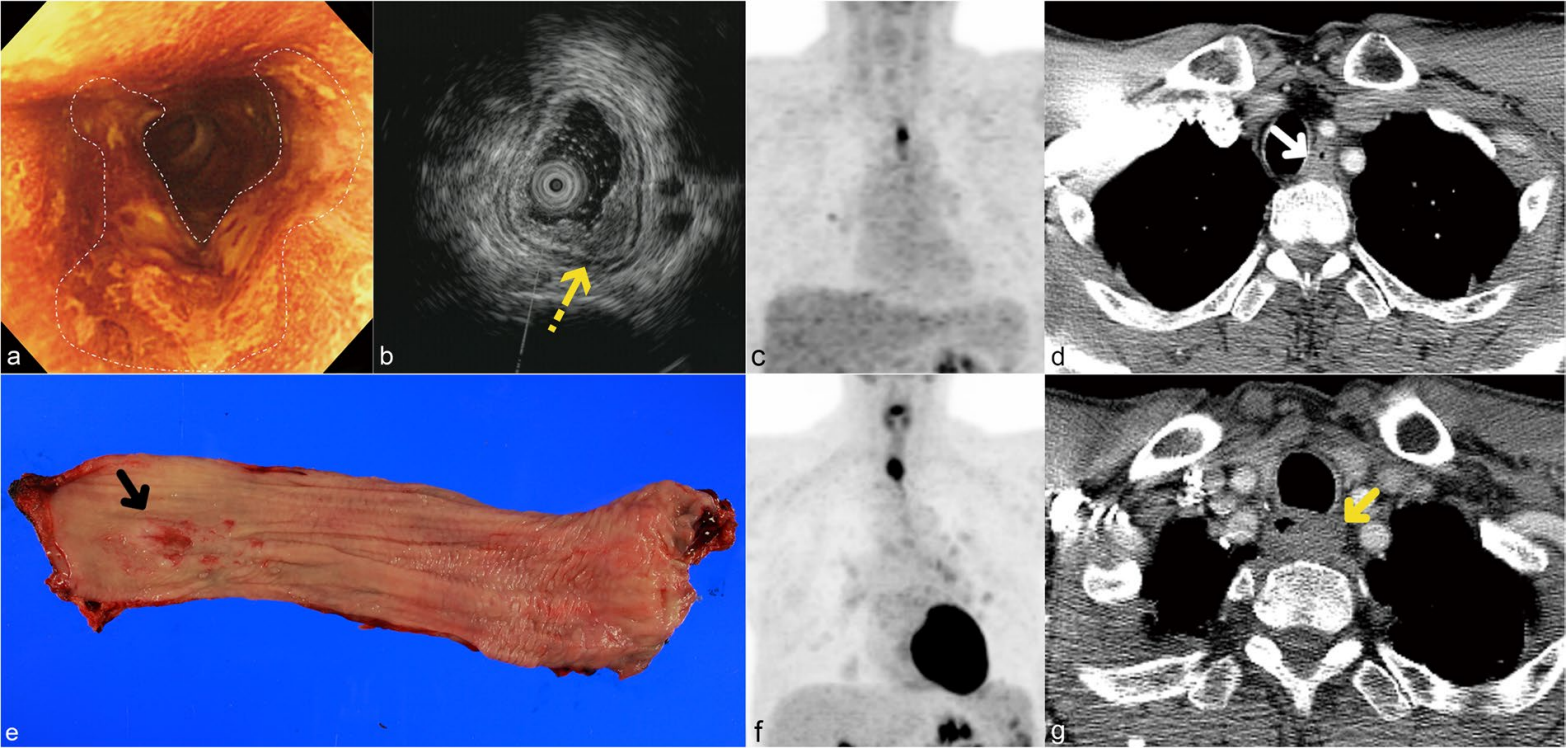
pT1bN0-stage esophageal squamous cell carcinoma in a 56-year-old man involving the intrathoracic upper thoracic esophagus without lymph node metastasis and R1 resection. (**a**) Chromoendoscopy with lugol shows a 3.0-cm-sized geographic lugol voiding lesion with an uneven surface (area in the white dashed line) in the upper thoracic esophagus (22 cm from the incisor teeth). (**b**) Endoscopic ultrasonography (EUS) showing the tumor invading the submucosal layer (yellow dotted arrow). (**c**) Maximum intensity projection (MIP) positron emission tomography (PET)/computed tomography (CT) scan demonstrating a hypermetabolic lesion (SUV_max_ = 6.7 at the presumed tumor site, favoring T2 stage) at the upper thoracic esophagus where the abnormality was seen on endoscopic ultrasonography. (**d**) Transverse contrast-enhanced computed tomography (CT) scan showing concentric wall thickening (white arrow) in the upper intrathoracic esophagus with a posterior wall thickness of 9.5 mm favoring T1b stage. (**e**) Gross specimen showing a 4.0-cm-length superficially depressed tumor (black arrow) histopathologically confirmed as esophageal squamous cell carcinoma with submucosal invasion (pT1b). (**f**) Follow-up maximum intensity projection positron emission tomography (PET)/computed tomography (CT) image obtained 23.4 months after a transthoracic esophagectomy, and three-field lymph node dissection demonstrating a new hypermetabolic lesion at the level of anastomosis (local recurrence). (**g**) Follow-up axial computed tomography (CT) image showing concentric wall thickening (yellow arrow) at the level of anastomosis, further confirming a local recurrence.

Among patients who experienced recurrence, there were three patients with an isolated local tumor recurrence (Fig. 1), 48 patients with isolated regional lymph node metastasis, eight patients with both local recurrence and regional lymph node recurrence, 14 patients with regional lymph node recurrence and distant metastasis, 12 patients with distant metastasis, three patients with local tumor recurrence, regional lymph node metastasis and distant metastasis, and one patient with local tumor recurrence and distant metastasis. Among the 45 metastases in the 30 patients with distant metastasis, the most common sites of metastasis were the lungs (n = 13), liver (n = 12), bone (n = 9), pleura (n = 4), thyroid glands (n = 2), adrenal glands (n = 2), spleen (n = 1), ureter (n = 1), and soft tissue (n = 1).

### Relationships among SUV_max_ and tumor characteristics, T, or N stage

SUV_max_ was significantly correlated with both pathologic T stage and the presence of nodal metastasis in a linearly positive fashion (r = 0.536, *p* < 0.001; r = 0.282, *p* < 0.001; respectively). However, there was no significant correlation between SUV_max_ and tumor differentiation (*p* = 0.520). SUV_max_ was also significantly correlated with pathologic N stage in a linearly positive fashion (r = 0.313, *p* < 0.001). Tumor size, defined as the maximum diameter of the tumor in a pathological specimen, was also significantly linearly correlated with SUV_max_ (r = 0.342, *p* < 0.001).

### Validation of the diagnostic performance of preoperative T staging with PET/CT

There were no significant differences in terms of the demographics or tumor characteristics of patients in the validation group and the original experimental group. Details of patients’ characteristics are described in Table 2.

**Table 2:** Patient Demographics and Tumor Characteristics of the Validation Group.

Characteristics	Group		
	Validation	Original	*p-* values*
Sex			0.846
Male	105	403	
Female	9	32	
Mean age (yrs)**	64	64	0.387
(min–max)	(31–90)	(31–90)	
Pathologic T stage			0.182
Tis + T1a	29	131	
T1b	72	234	
T2	13	70	
Tumor length (cm)	2.35	2.36	0.195
(Q1–Q3)	(1.50–3.00)	(1.50–3.00)	
Tumor differentiation			0.007
G1	5	68	
G2	93	308	
G3	16	59	
Adjuvant Tx			0.969
Yes	20	77	
No	94	358	
R0 resection			0.966
Yes	113	431	
N0	1	4	
Tumor Locations			0.298
Cervical	1	1	
Upper thoracic	12	35	
Mid thoracic	50	169	
Lower thoracic	36	189	
Upper to mid thoracic^†^	4	9	
Mid to lower thoracic^†^	10	28	
Upper to lower thoracic^†^	1	2	
Upper and lower thoracic^††^	0	2	
Nodal metastasis			0.481
No	82	298	
Yes	32	137	
Total	114	435	

Note. ^__^ *Calculated using the Chi-square test, Q = quartile (Q1–25 percent quartile, Q3–75 percent quartile); G1 = well-differentiated, G2 = moderately differentiated, G3 = poorly differentiated; Definition of the tumor location; Cervical = superior to the thoracic inlet, Upper thoracic = from the thoracic inlet to the azygos arch, Middle thoracic = from the azygos arch to the inferior pulmonary veins, Lower thoracic = from the inferior pulmonary veins to the esophagogastric junction; Upper to middle thoracic^†^ = long segment involvement from the upper to middle esophagus, Mid to lower thoracic^†^ = long segment involvement from the middle to lower esophagus, Upper to lower thoracic^†^ = long segment involvement from the upper to lower esophagus; Upper and lower thoracic^††^ = the upper and lower thoracic esophagus skipping the middle thoracic esophagus.

For differentiating ≤T1a from other eSCCs, the sensitivity, specificity, accuracy, positive predictive value (PPV), and negative predictive value (NPV) of PET/CT in the validation group were 51.7% (16/29, 95% confidence interval [CI]; 32.5–70.6%), 82.4% (71/85, 95% CI; 73.9–90.75%), 75.4% (86/114, 95% CI; 66.5–83.0%), 51.7% (15/29) and 83.5% (71/85), respectively, whereas those in the experimental study group were 74.8% (98/131, 95% CI; 66.5–82.0%), 70.1% (213/304, 95% CI; 64.6–75.2%), 71.5% (311/435, 95% CI; 67.0–75.7%), 51.9% (98/189) and 86.6% (213/246), respectively.

### Prognostic significance of SUV_max_ with survival and recurrence

In the receiver operating characteristic (ROC) curve analysis, a cut-off value of SUV_max_ 3.05 and 5.65, respectively, was the most useful for differentiating <T1a eSCCs from other cancers and for differentiating T1 (<T1b) eSCCs from T2 eSCCs^9^.

**Figure 2:**
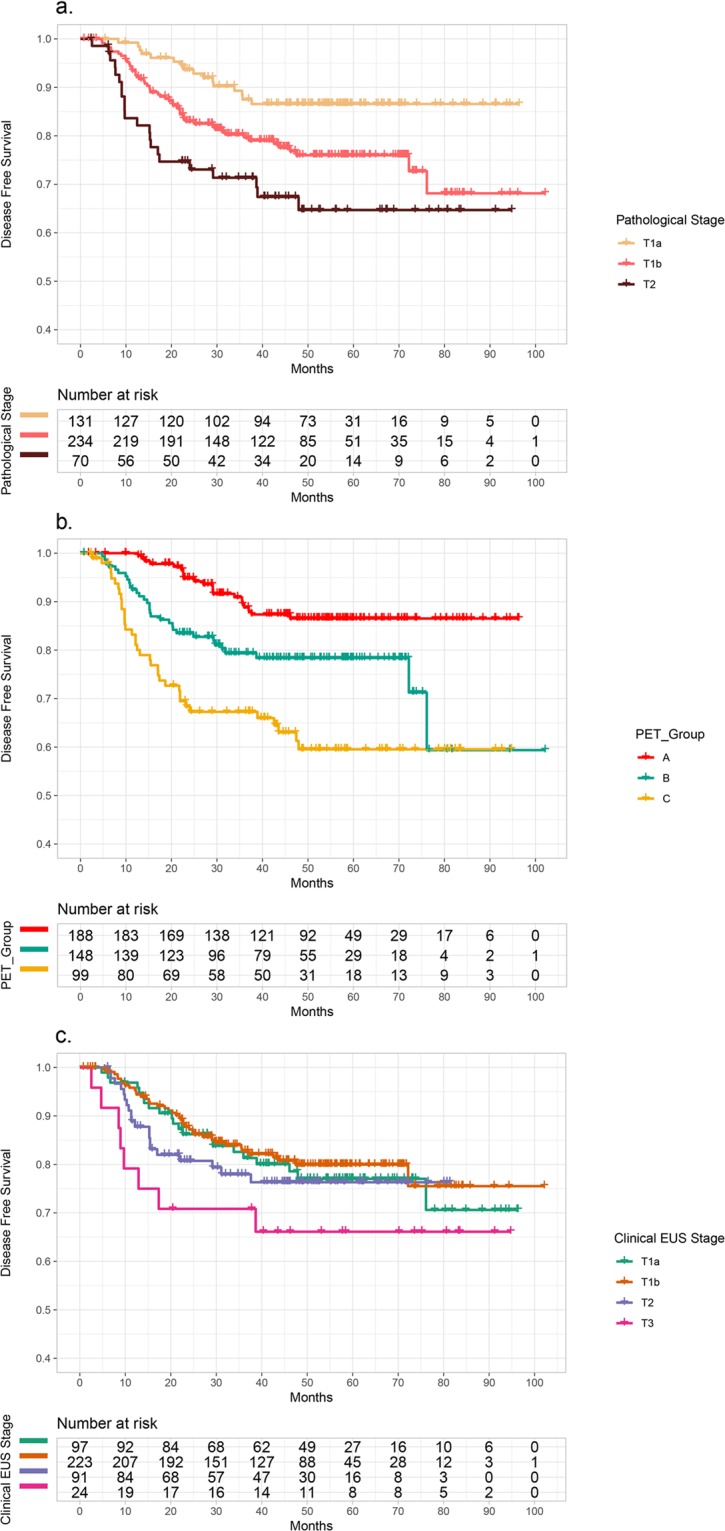
Kaplan-Meier plots of disease-free survival (DFS) based on (**a**) pathological T stage (*p* < 0.001), (**b**) SUV_max_ (A: SUV_max_ ≤ 3.05, B: SUV_max_ 3.06–5.64, C: SUV_max_ ≥ 5.65) and (**c**) clinical T stage by endoscopic ultrasonography (EUS).

**Figure 3:**
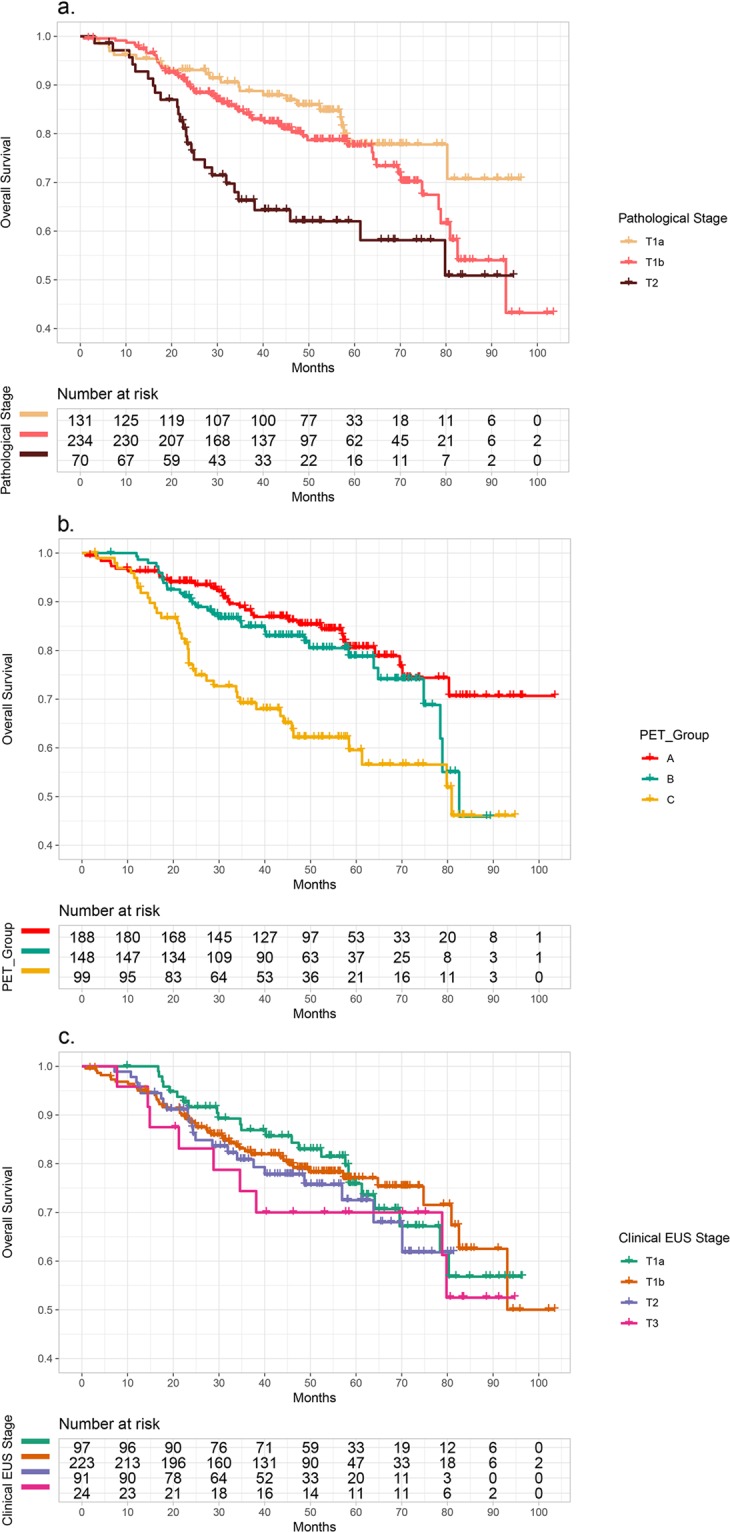
Kaplan-Meier plots of overall survival (OS) based on (**a**) pathological T stage (*p* < 0.001), (**b**) SUV_max_ (A: SUV_max_ ≤ 3.05, B: SUV_max_ 3.06–5.64, C: SUVmax ≥ 5.65) and (**c**) clinical T stage by endoscopic ultrasonography (EUS).

By applying these SUV_max_ cut-off values, we statistically proved that both DFS and OS were significantly different among the three groups when divided by the cut-off values (A: SUV_max_ ≤ 3.05, B: SUV_max_ 3.06–5.64, C: SUV_max_ ≥ 5.65), except the OSs between groups A and B (DFS, *p* < 0.001; A vs. B, *p* = 0.005; A vs. C, *p* < 0.001; B vs. C, *p* = 0.010; OS, *p* < 0.001; A vs. B, *p* = 0.167; A vs. C, *p* < 0.001; B vs. C, *p* = 0.009; Figs. 2 and 3). The five-year DFS was 86.5% in group A, 78.4% in group B, and 59.5% in group C (Table 3). The five-year OS was 80.6% in group A, 78.7% in group B, and 59.5% in group C (Table 4). However, clinical T stage, determined by EUS, was not statistically different between the groups in terms of DFS or OS (DFS: p = 0.324, OS: *p* = 0.753) (Figs. 2 and 3).

**Table 3:** Univariate and Multivariate Analyses Evaluating Disease-Free Survival.

Variable	Univariate Analysis*			Multivariate analysis**			
				Model 1 using PET staging		Model 2 using pathologic T staging	
	5-year DFS	χ^2^	*p-* value	HR (95% CI)	*p-* value	HR (95% CI)	*p-* value
Sex		3.929	**0.047**		**0.040**		0.073
Male	76.3%			Reference		Reference	
Female	93.4%			0.23 (0.06–0.94)		0.28 (0.07–1.13)	
Age		0.893	0.345	Not included		Not included	
<60	74.2%						
≥60	79.0%						
R0 resection		0.106	0.744	Not included		Not included	
Yes	77.6%						
No	75.0%						
Adjuvant Tx		16.976	**<0.001**		0.389		0.991
No	81.6%			Reference		Reference	
Yes	59.6%			1.29 (0.72–2.30)		1.00 (0.54–1.85)	
Locations		3.749	0.711	Not included		Not included	
Tumor length***		1.295	0.255	Not included		Not included	
<2.5 cm	79.0%						
≥2.5 cm	76.2%						
Tumor differentiation		2.052	0.358	Not included		Not included	
G1	82.4%						
G2	74.6%						
G3	71.7%						
Pathologic T stage		13.130	**0.001**	Not included			0.109
Tis + T1a	86.6%					Reference	
T1b	76.0%					1.46 (0.81–2.65)	
T2	64.7%					2.08 (1.05–4.14)	
Nodal metastasis		23.693	**<0.001**		**0.035**		**0.022**
No	84.1%			Reference		Reference	
Yes	63.0%			1.82 (1.04–3.16)		1.94 (1.10–3.43)	
Clinical T stage (EUS)		3.476	0.324	Not included		Not included	
Less than T1a	77.0%						
T1b	80.0%						
T2	76.3%						
T3	66.1%						
PET staging group		28.415	**<0.001**		**<0.001**	Not included	
A: SUV_max_ ≤ 3.05	86.5%			Reference			
B: SUV_max_ 3.06–5.64	78.4%			1.86 (1.07–3.25)			
C: SUV_max_ ≥ 5.65	59.5%			3.24 (1.85–5.66)			

Note—*Calculated using the log-rank test of the differences between the two survival curves generated using the Kaplan-Meier curve; **Calculated using the Multivariate Cox proportional hazard model; ***Groups by tumor length were divided using a cut-off 2.5 cm close to the mean value of tumor length.

EUS: Endoscopic ultrasonography; PET: positron emission tomography; SUV_max_: maximum standardized uptake value; CI: confidence interval.

**Table 4:** Univariate and Multivariate Analyses Evaluating Overall Survival.

Variable	Univariate Analysis*			Multivariate analysis**			
				Model 1 using PET staging		Model 2 using pathologic T staging	
	5-year OS	χ^2^	*p-*value	HR (95% CI)	*p-*value	HR (95% CI)	*p-* value
Sex		3.482	**0.062**		0.053		0.080
Male	74.2%			Reference		Reference	
Female	88.7%			0.322 (0.10–1.02)		0.36 (0.11–1.13)	
Age		0.524	0.469	Not included		Not included	
<60	79.0%						
≥60	73.5%						
R0 resection		0.017	0.896	Not included		Not included	
Yes	75.1%						
No	75.0%						
Adjuvant Tx		2.511	0.113	Not included		Not included	
No	76.0%						
Yes	71.3%						
Locations		4.337	0.631	Not included		Not included	
Tumor length***		0.373	0.542	Not included		Not included	
<2.5 cm	75.9%						
≥2.5 cm	74.1%						
Tumor differentiation		2.161	0.339	Not included		Not included	
G1	82.4%						
G2	73.6%						
G3	71.7%						
Nodal metastasis		5.435	**0.020**		0.177		0.144
No	77.0%			Reference		Reference	
Yes	70.9%			1.33 (0.88–1.99)		0.73 (0.48–1.11)	
Pathologic T stage		11.488	**0.003**	Not included			**0.022**
Tis + T1a	77.8%					Reference	
T1b	77.6%					1.25 (0.75–2.09)	
T2	62.0%					2.18 (1.20–3.94)	
Clinical T stage (EUS)		1.198	0.753	Not included		Not included	
Less than T1a	75.9%						
T1b	77.1%						
T2	72.5%						
T3	70.0%						
PET staging group		17.367	**<0.001**		**<0.001**	Not included	
A: SUV_max_ ≤ 3.05	80.6%			Reference			
B: SUV_max_ 3.06–5.64	78.7%			1.38 (0.85–2.25)			
C: SUV_max_ ≥ 5.65	59.5%			2.66 (1.66–4.25)			

Note ^__^ * Calculated using the log-rank test of the differences between the two survival curves generated using the Kaplan-Meier curve; **Calculated using the Multivariate Cox proportional hazard model; ***Groups by tumor length were divided using a cut-off 2.5 cm close to the mean value of the tumor length.

EUS: Endoscopic ultrasonography; PET: positron emission tomography; SUV_max_: maximum standardized uptake value; CI: confidence interval.

In the univariate analysis, sex, adjuvant therapy, nodal metastasis, SUV_max_, and pathological T stages were significant prognostic factors for tumor recurrence. SUV_max_, the presence of nodal metastasis, and pathological T stage were significant prognostic factors for survival. As the cut-off values of SUV_max_ were extracted by pathological T stage groups, we assumed two different models for the multivariate analysis using pathological T stage and hypothetical stages using SUV_max_ (PET stage groups, Tables 3 and 4). This was despite the severity of multicollinearity not being significant between those with pathological T stage and hypothetical stage as determined by the SUV_max_ (variance inflation factor [VIF] = 1.594). In the multivariate analysis, SUV_max_ and the presence of nodal metastasis were significant factors for disease recurrence. SUV_max_ and pathological T stage were significant factors for patient survival (Tables 3 and 4; Figs. 2 and 3).

### Comparison of the prognostication performance of each staging system

For predicting DFS, the iAUC value of hypothetical staging using SUV_max_ was 0.645. This was greater than those of other staging systems at all time points (pathological T staging, 0.598; clinical T staging using EUS, 0.533). For predicting the OS, the iAUC value of hypothetical staging using SUV_max_ was 0.602. This was also greater than those of other staging systems at all time points (pathological T staging, 0.580; clinical T staging using EUS, 0.525).

## Discussion

It is known that with surgically resected eSCCs, the pathological stage is an independent risk factor for recurrence within the first year after surgery and that the presence of lymph node metastasis is the most common relapse pattern after an esophagectomy^10^.

In our study, the SUV_max_ on PET/CT was also observed as an independent factor for predicting both future recurrences and patient survival, along with the pathologic T stage (for predicting survival) and nodal metastasis presence (for disease recurrence). Additionally, the SUVmax was positively correlated with both pathological T and N stages and tumor length.

There have been a few studies published recently regarding the value of measuring SUV_max_ on PET/CT as a prognostic factor in eSCCs. These studies showed similar results to those seen in this study. According to Jeon *et al*.^11^, venous invasion and high SUV_max_ could be important prognostic factors for disease recurrence in T1N0M0 eSCCs. Song *et al*. also reported similar results from their cohort in which all stages of eSCCs were included^12^. Our results were derived from a patient cohort, included a larger study population. And our study had a longer follow-up period than other studies. Moreover, we performed a more comprehensive analysis to look at predicting pathological T stage and also survival using the SUV_max_ of primary tumors.

In the Kaplan-Meier curve analyses for both DFS and OS based on the SUV_max_ cut-off, two survival curves of groups B and C crossed over each other at the time point of 65 months after surgery (Figs. 2b and 3b). The same phenomenon was also seen in the Kaplan-Meier curve for OS based on the pathologic T staging (Fig. 3a). This may have resulted from a small number of samples whose follow-up periods were greater than 70 months. The 95% CIs of group B for both DFS and OS widened rapidly 70 months after surgery (Supplemental Fig. 2).

The usefulness of FDG PET/CT for tumor staging or prognostication in esophageal cancer has been unclear, particularly in patients with early-stage (T1) cancers and in esophageal adenocarcinomas. Cuellar *et al*.^13^ asserted that FDG PET/CT is not useful in evaluating adenocarcinoma of the esophagus when endoscopic biopsy discloses Tis and T1 in tumor stage. They believed that because regional nodal metastases are uncommon and distant metastases are rare in patients with T1-stage esophageal cancers as well as because FDG PET/CT can lead to inappropriate management, FDG PET/CT should not be used in evaluating patients with clinical Tis and T1 esophageal adenocarcinomas. Contrastingly, in our study, in which eSCCs of T1a, T1b, and T2 were included, measuring SUV_max_ appeared to help differentiate the T stages and in predicting oncoming recurrent disease (DFS) and patient survival (OS) in patients who had a surgically resected eSCC.

Reduced spatial and contrast resolutions of PET/CT were considered one of the interpretative downsides of using PET/CT for T-descriptor^14^. However, by using SUV_max_ of the primary tumor rather than the visualized gross tumor volume, PET/CT can help differentiate pathologic T stages. In our previous study, measuring the SUV_max_ of primary cancer was shown to assist in differentiating <T1a from T1b or T2 cancers and between <T1 and T2 cancers^9^.

In our study, the cut-off value of SUV_max_ 3.05 was effective in discriminating T1a-stage from T1b- or T2-stage eSCCs. Similarly, Furukawa *et al*.^15^ showed that FDG-PET helped to diagnose tumors in 40 consecutive cT1N0M0 eSCC patients involving the submucosa but not beyond the middle one-third of the submucosa (SM2) and beyond, or tumors having occult lymph node metastasis. Their proposed optimal cut-off SUV_max_ value of 2.7 can be used distinguishing ESD candidate patients from advanced stage eSCC patients with an SM2 involvement and beyond (21, 52.5%) or lymph node metastasis (6, 15%). Our optimized cut-off value SUV_max_ 3.05 was slightly higher, partly because we tried to discriminate eSCCs of T1a or less from higher-stage cancers. It could also be because we used a larger study population (435 eSCC patients). The pathologic T1b stage includes any tumors involving the submucosa (SM1, SM2, and SM3).

In a prospective validation study using different patient cohorts, PET/CT, and the SUVmax of the tumor showed its efficaciousness in triaging patients with various T stages. Furthermore, in the current study, hypothetical T stages determined with the measured SUV_max_ regarding the primary tumor appear to be more practical and accurate in predicting patients’ prognoses than those determined with EUS. When a time-dependent AUC metric was used, the prediction model adopting the hypothetical T sage on disease recurrence and survival excelled the other models adopting pathologic T stage and clinical T stage by using EUS. The SUV_max_ of the tumor positively correlated with the depth of tumor invasion (pathologic T stage) and also with the tumor length and nodal metastasis (pathologic N stage). The pathologic T stage or clinical T stage (determined by the use of EUS) might not have reflected the relationship with nodal metastasis.

It is known that the length of eSCC has a positive correlation with the SUV_max_ of the primary tumor^16,17^. In a study by Xu *et al*.^18^, the tumor length demonstrated a positive correlation with tumor recurrence. However, in the present study, the tumor length was not an independent prognostic factor in surgically resectable eSCCs.

Our study had several limitations. First, it was conducted in a single tertiary referral hospital. Second, our study population was retrospectively recruited from our surgical registry, and the patients had a resectable early-stage (only T1 and T2-stage) eSCC. This may have contributed to selection bias. Third, we included patients who underwent both CT and PET/CT at our institution. Thus the measurement methods of SUV_max_ and CT and PET/CT study protocols were uniformly standardized. Therefore, the results of our study may not be generalized for patients with eSCC worldwide. However, we tried to include a large number of early-stage eSCC patients. Further studies with a multi-centered prospective design and with a larger number of patients may be needed to validate our study results. Fourth, we excluded patients who underwent neoadjuvant chemotherapy. Future studies including patients undergoing neoadjuvant chemo- or radiation therapy may be needed to assess the effectiveness of measuring SUV_max_ on PET/CT in eSCC patients. Lastly, we did not consider the possible effect of the angiolymphatic or perineural invasion of the primary tumor in the survival analysis. This was because there were only a few identified angiolymphatic or perineural invasion cases in our patient cohort.

In conclusion, of the 435 patients undergoing surgical resection with a T1- or T2-stage eSCC, 103 (23.7%) patients died, and 89 (20.5%) patients experienced tumor recurrence during the median follow-up period of 49.3 months (range; 0.8 to 105 months). In the multivariate analysis, the SUV_max_ of the primary tumor (eSCC) on PET/CT was an independent factor for predicting future recurrences and patient survival. The pathologic T stage was a predictor for survival, and nodal metastasis was a predictor for recurrence. Furthermore, the SUV_max_ was significantly correlated with both pathological T and N stages in a linearly positive manner. Therefore, in patients with a surgically resectable eSCC, measuring the SUV_max_ of the primary tumor could help predict patient survival.

## Materials and Methods

### Study population and data collection

**Figure 4:**
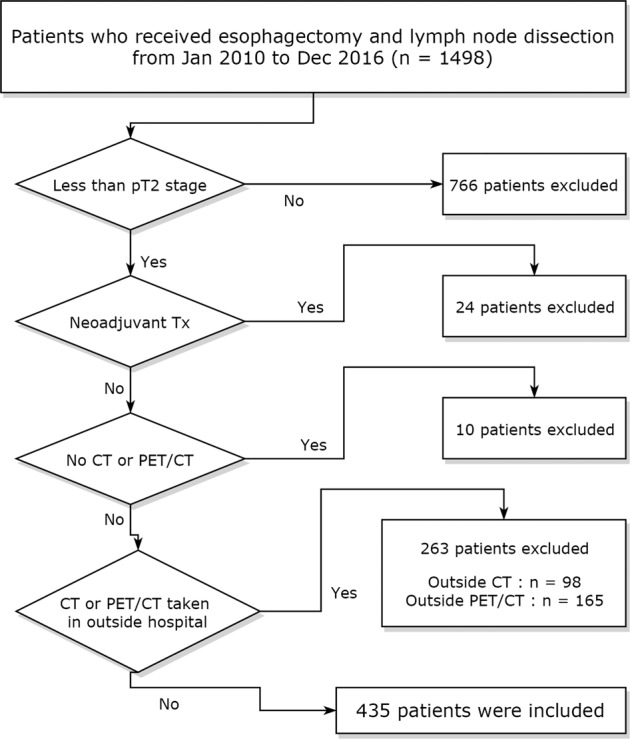
Workflow of the experimental study design. PET: positron emission tomography; CT: computed tomography.

Using the Esophageal Cancer Surgery Registry at the Samsung Medical Center (a 1,979-bed tertiary referral hospital in Seoul, South Korea), 1498 patients who received esophagectomy and lymph node dissection were identified between January 2010 and December 2016. Of them, 732 patients had <T1a, T1b, or T2 stage eSCCs. Among them, 297 patients were excluded for the following reasons: 24 patients were excluded due to having undergone neoadjuvant chemoradiation therapy (*n* = 7) or concurrent chemoradiation therapy (*n* = 17), 10 patients did not undergo either enhanced chest CT or PET/CT, 165 patients had their PET/CT in outside hospital (difficulty in measuring SUV_max_), and 98 patients had their chest CT in an outside hospital (with incomplete or different CT parameters for evaluating ECA and its staging). The remaining 435 patients who underwent both PET/CT and chest CT at our institution were included in this study^9^ (Fig. 4).

For the validation study of FDG-PET/CT as a possible T descriptor, we also found 224 patients who received an esophagectomy and lymph node dissection between January 2017 and December 2018. Of these, 110 patients were excluded for the following reasons: 50 were more than T3 stage, 38 had undergone their PET/CT in an outside hospital, and 22 had undergone surgery for recurrent ECA. Thus, 114 patients were included in the validation study, where we prospectively performed T staging using the SUV_max_ criteria acquired with an experimental study. The diagnostic performance of FDG-PET/CT for a T descriptor was compared with that of the experimental study.

Patient-related (age, sex, adjuvant chemotherapy, and survival), surgery-related (type of surgery, and surgical resection margin)-, and tumor-related (length, location, histology, and pathologic stage) factors were collected from the database. Details of patients’ surgeries and their pathologic specimen analyses are described in our previously published article^9^.

The Institutional Review Board (IRB) of the Samsung Medical Center approved this retrospective study (IRB no. 2017–04–093). Informed consent for reviewing patients’ electronic medical records was waived by the IRB. Our study design, data collection, and analysis were performed as per the relevant guidelines and regulations.

### Preoperative PET/CT scanning and Interpretation

All patients fasted for at least 6 hours before their PET examination. Blood glucose levels were measured before the injection of FDG and were required to be <200 mg/dL in all patients. Whole-body PET and unenhanced CT images were acquired using two types of PET/CT scanners (Discovery LS, GE Healthcare, Milwaukee, WI, USA; Discovery STe, GE Healthcare, Milwaukee, WI, USA), 60 minutes after the injection of FDG (5.5 MBq/Kg). When the Discovery STe scanner was used, a whole-body CT was performed using a continuous spiral technique with a 16-slice helical CT (140 keV; 30–170 mA; section width, 3.75 mm). After the CT scan, an emission scan was obtained from the head to middle thigh for 2.5 min per frame in a 3-dimensional mode. Attenuation-corrected PET images (3.9 × 3.9 × 3.3 mm) were reconstructed from the CT data using an ordered-subset expectation maximization (OSEM) algorithm (20 subsets, 2 iterations). When the Discovery LS scanner was used, a whole-body CT was performed using a continuous spiral technique with an 8-slice helical CT (140 keV; 40–120 mA; section width, 5 mm). After the CT scan, an emission scan was obtained from the head to middle thigh for 4 min per frame in a 2-dimensional mode. Attenuation-corrected PET images (4.3 × 4.3 × 3.9 mm) were reconstructed from the CT data using an OSEM algorithm (28 subsets, 2 iterations). The standardized uptake value (SUV) was derived from the injected dose of FDG, and the patient’s body weight^9^.

One of the two nuclear medicine physicians (16 years and 10 years of experience in PET/CT interpretation, respectively) and one chest radiologist (26 years of chest CT interpretation and 10 years of PET/CT interpretation), who were blind to the clinical and pathologic results, evaluated the PET/CT in consideration of the chest CT results. When identifiable esophageal lesions were present, the location was recorded with four anatomic landmarks used for categorization: thoracic inlet, azygos arch, inferior pulmonary veins, and the esophagogastric junction.

As for T staging, the SUV_max_ was measured at the tumor sites. When the primary cancer was not visualized or could not be distinguished from the background (*n* = 70), the SUV_max_ was assigned an assumed default value of 1.0, similar to the background uptake.

In our previous study, the ROC curves were constructed and depicted to obtain the most appropriate cut-off values in terms of differentiating <T1a from T1b or T2 and differentiating <T1 and T2^9^. We divided patients into three hypothetical PET staging groups A, B and C using two cut-off values of SUV_max_ 3.05 and 5.65: A = SUV_max_ < 3.05, SUV_max_ 3.05 < B < SUV_max_ 5.65, and C = SUV_max_ ≥ 5.65, respectively.

### Patient surveillance

Patients in this retrospective cohort were followed up regularly following the specific surveillance protocols at our institution after surgery. Specifically, out-patient based clinic appointments were arranged every 6 months at our institution for 5 years. After 5 years, patients were usually referred to a secondary referral hospital in their hometown. Follow-up imaging studies were also performed at specific intervals: a chest x-ray every month for the first 2 months to check for complications after surgery, a contrast-enhanced chest CT scan every 6 months for 5 years, a PET/CT and esophagogastroduodenoscopy every year, or at any time when symptoms indicated a recurrence had occurred.

Overall survival (OS) was defined as the length of time from either the date of diagnosis or the start of treatment to death. Disease-free survival (DFS) was defined as the diagnosis of a locoregional recurrence, including lymph node metastasis or distant metastasis at any site. The closing date for survival data collection was December 31, 2018, which was 2 years after the surgery of the most recently enrolled patients in our cohort.

### Statistical analysis

Pearson’s product-moment correlation coefficient was calculated for analyzing the relationship between the SUV_max_ of the primary eSCC and the pathologic T and N stages, the presence of nodal metastasis, or primary tumor histologic differentiation.

Five-year OS and DFS were calculated and plotted using the Kaplan-Meier method. Differences in survival among the three (T1a, T1b, and T2 stages) groups were assessed by the three staging methods (pathologic, clinical [EUS], and hypothetical PET by using the SUV_max_) were compared using the log-rank test. Multivariate Cox proportional hazard models for OS and DFS were built for those prognostic factors with a *p*-value of <0.1 in the univariate analysis. For detecting multi-co-linearity among the prognostic factors used for the multivariate analysis, VIFs were calculated.

To evaluate the predictive value for survival (prognostication performance) among the three staging systems, including pathologic T staging, clinical staging using EUS, and hypothetical PET staging, we used an integrated time-dependent AUC (iAUC) calculated from time 0 to 60 months after surgery^19^.

For the validation of SUV_max_ as a T descriptor, we calculated the sensitivity, specificity, accuracy, PPV, and NPV, and compared the results with those of the experimental study. To see whether there were demographic differences between the experimental and validation groups, the data were analyzed using the chi-square test.

All statistical analyses were done using SPSS (SPSS for Windows, version 22.0; SPSS, Chicago, IL) and the statistical computing language R (version 3.4.3, R Foundation). A *p-*value of <0.05 was considered statistically significant.

## Supplementary information

Supplementary Information.

## Data Availability

The datasets generated during and/or analyzed in the current study are available from the corresponding author on reasonable request.
