# Supplementary material for: Surgically Resected Esophageal Squamous Cell Carcinoma: Patient Survival and Clinicopathological Prognostic Factors
Source: Sci Rep. 2020 Mar 19;10:5077. doi: 10.1038/s41598-020-62028-5 (PMC7081270; doi:10.1038/s41598-020-62028-5)
Supplement: Supplementary file 1 — Supplementary Information. [file 41598_2020_62028_MOESM1_ESM.docx]

Supplementary Information

**Surgically Resected Esophageal Squamous Cell Carcinoma: Patient Survival and Clinicopathological Prognostic Factors**

Dong Young Jeong, MD^1^ **^.^** Kyung Soo Lee, MD^1^ **^.^** Joon Young Choi, MD^2^ **^.^**

Myung Jin Chung, MD^1^ **^.^** Yang Won Min, MD^3^ **^.^** Hong Kwan Kim, MD^4^ **^.^** Jae Ill Zo, MD^4^ **^.^** Young Mog Shim, MD^4^ **^.^** Jong-Mu Sun, MD^5^

^1^Department of Radiology, Samsung Medical Center, Sungkyunkwan University School of Medicine (SKKU-SOM), Seoul, South Korea;
^2^Department of Nuclear Medicine, Samsung Medical Center, Sungkyunkwan University School of Medicine (SKKU-SOM), Seoul, South Korea; ^3^Division of Gastroenterology, Department of Medicine, Samsung Medical Center, Sungkyunkwan University School of Medicine (SKKU-SOM), Seoul, South Korea;
^4^Department of Thoracic Surgery, Samsung Medical Center, Sungkyunkwan University School of Medicine (SKKU-SOM), Seoul, South Korea;
^5^Division of Hemato‐oncology, Department of Medicine, Samsung Medical Center, Sungkyunkwan University School of Medicine (SKKU-SOM), Seoul, South Korea;


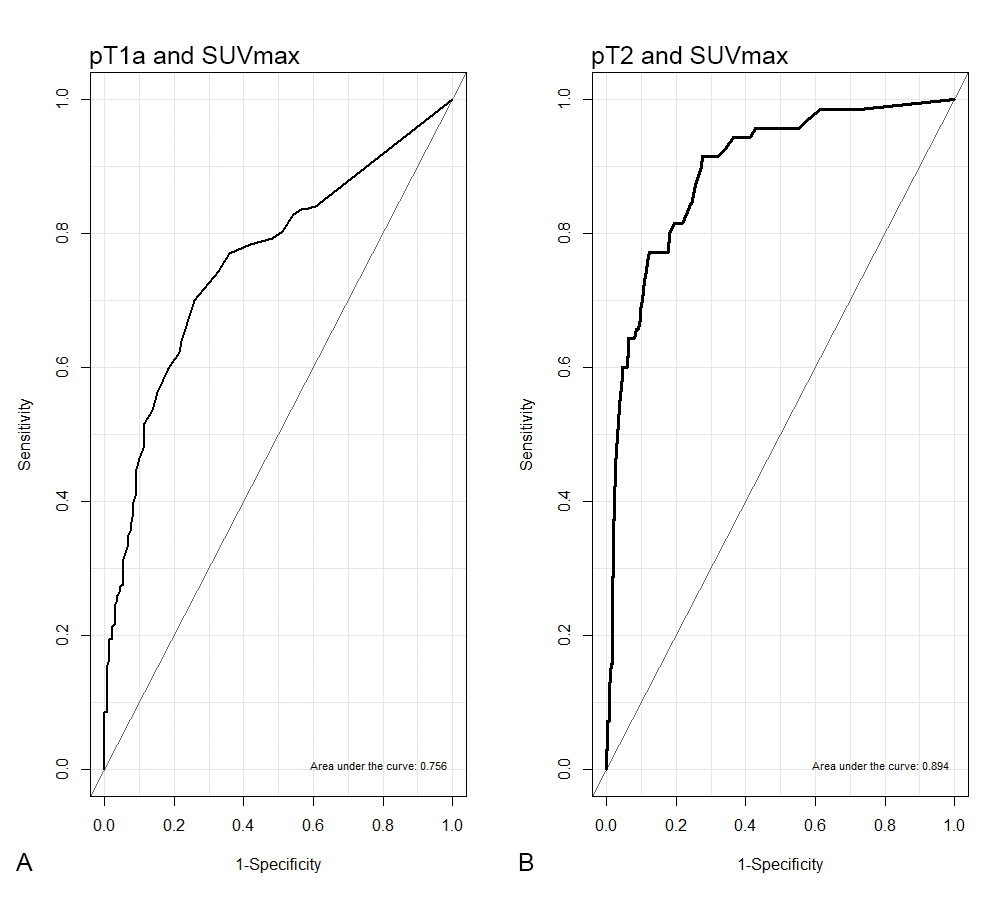


**Supplementary Figure 1.** ROC Curve Analysis of SUV_max_ of Primary Esophageal Cancer

With ROC curve analysis, cut off value of SUV_max_ 3.05 (AUC: 0.757; 95% CI, 0.710–0.803; *P* < .001) at PET provided sensitivity 74.8% (98/131), specificity 70.1% (213/304), accuracy 71.5% (311/435), PPV 51.9% (98/189) and NPV 86.6% (213/246), respectively, for differentiating < T1a eSCCs from other cancers. Cut off value of SUV_max_ 5.65 (AUC: 0.897; 95% CI, 0.857–0.937; *P* < .001) provided sensitivity 77.1% (54/70), specificity 87.7% (320/365), accuracy 86.0% (374/435), PPV 54.5% (54/99) and NPV 95.2% (320/336), respectively, for differentiating T1 (< T1b) eSCCs from T2 eSCCs^1^.

**
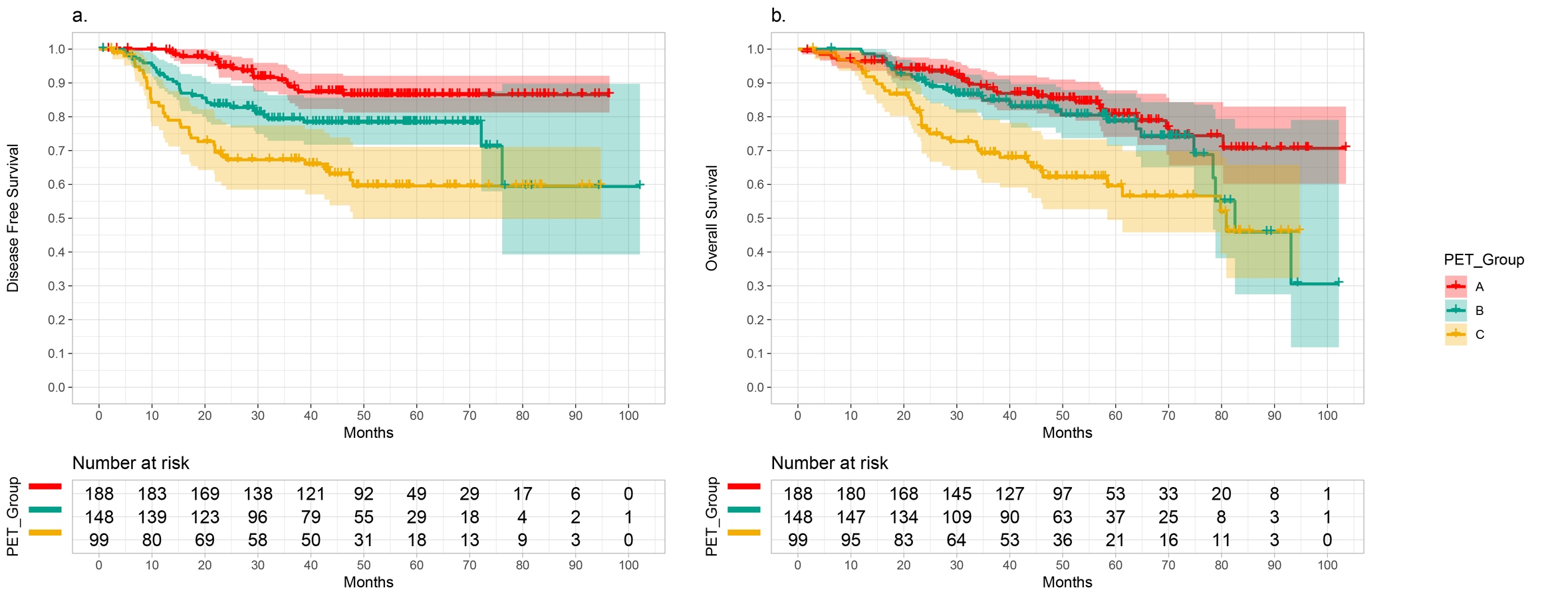
**

**Supplementary Figure 2.** Detailed Kaplan-Meier Curves, Based on Three Groups Using SUVmax Cut-off, of both (a) Disease Free Survival and (b) Overall Survival with 95% Confidence Intervals

**Reference**

1. Jeong, D. Y. *et al.* Surgically resected T1- and T2-stage esophageal squamous cell carcinoma: T and N staging performance of EUS and PET/CT. *Cancer Med* **7**, 3561-3570 (2018).
